# Association between triglyceride glucose index and peak growth hormone in children with short stature

**DOI:** 10.1038/s41598-021-81564-2

**Published:** 2021-01-21

**Authors:** Qianqian Zhao, Yuntian Chu, Hui Pan, Mei Zhang, Bo Ban

**Affiliations:** 1 Department of Endocrinology, Qingdao University, Qingdao, 266071 Shandong People’s Republic of China; 2 Department of Endocrinology, Affiliated Hospital of Jining Medical University, Jining Medical University, 89 Guhuai Road, Jining, 272029 Shandong People’s Republic of China; 3 School of Health Management and Medicine, Tongji Medical College, Huazhong University of Science and Technology, Wuhan, 430030 Hubei People’s Republic of China; 4 Key Laboratory of Endocrinology of National Health Commission, Department of Endocrinology, Peking Union Medical College Hospital, Chinese Academy of Medical Science and Peking Union Medical College, Beijing, 100730 People’s Republic of China; 5 Chinese Research Center for Behavior Medicine in Growth and Development, 89 Guhuai Road, Jining, 272029 Shandong People’s Republic of China

**Keywords:** Endocrine system and metabolic diseases, Risk factors

## Abstract

Growth hormone (GH) secretion is related to many factors, such as weight and puberty, and the reproducibility of GH provocation tests is very poor. This study aimed to evaluate whether the triglyceride (TyG) index was associated with peak GH in children with short stature. This study included 1095 children with short stature divided into two groups based on peak GH level in GH provocation tests [GH deficiency (GHD) group = 733 children; non-GHD group = 362 children]. We found that the TyG index was significantly higher in the GHD group than in the non-GHD group (*P* < 0.001). A nonlinear relationship was detected between the TyG index and peak GH, whose point was 7.8. A significant negative association between the TyG index and peak GH was observed when the TyG index was greater than 7.8 (β − 2.61, 95% CI − 3.98, − 1.24; *P* < 0.001), whereas, the relationship between the TyG index and peak GH was not significant when the TyG index was lower than 7.8 (β 0.25, 95% CI − 1.68, 2.17; *P* = 0.799). There is a nonlinear relationship between the TyG index and peak GH, and a higher TyG index is associated with decreased peak GH in children with short stature.

## Introduction

Short stature is defined by a height more than two standard deviation scores (SDS) below the median height for the relevant age and sex of the subject, and the most common reasons for short stature are growth hormone deficiency (GHD) and idiopathic short stature (ISS)^1^. Growth hormone (GH) provocation tests are indispensable in assessing the aetiology of short stature. However, GH secretion is regulated by multiple physiologic factors, including age, sex, body mass index (BMI) and onset of puberty, which sometimes may produce false-positive results^2–4^. Previous studies have demonstrated that obese children and adolescents have a reduced stimulated GH response to provocative testing^5^, and it is well known that there is a significant negative association between BMI and peak GH^6,7^. Furthermore, insulin resistance (IR) has been demonstrated to be involved in the relationship between BMI and reduced growth hormone secretion^8^. The triglyceride glucose (TyG) index, which is calculated from fasting measurements of triglycerides and glucose, has been recently suggested as a reliable surrogate marker of IR.

Considerable previous studies have demonstrated that the TyG index is significantly related to an increased risk of developing coronary artery disease (CAD), type 2 diabetes, prehypertension and hypertension^9–11^; among these conditions, IR plays a major role in their progression^12,13^. The hyperinsulinaemic-euglycaemic clamp is the “gold standard” test for measuring IR^14^. However, due to the complexity of the testing process, it is not commonly used in clinical settings. A previous study observed that the TyG index was more sensitive and specific for evaluating IR compared with the hyperinsulinaemic-euglycaemic clamp test^15^.

GH is a significant regulator of metabolic homeostasis by regulating liver uptake of triglycerides and glucose and stimulating lipolysis in adipose tissue^16^. Glucose and lipid abnormalities have been reported in patients with GHD^17^. A study has shown that GH regulates the body’s sensitivity to insulin as a direct result of its induction of lipid metabolism (release of free fatty acids) in adipose tissue^18^. IR may play an important role in glucose and lipid metabolism disorders associated with GHD. However, the association between IR and GH is controversial, with both positive and negative relationships reported^19–21^. Furthermore, the association of BMI and GH is well accepted; however, the potential mechanisms that underpin these effects are still not firmly established. It is well known that IR is closely related to obesity^22^ and that with an increase in BMI, GH secretion is reduced, indicating that IR is associated with the level of GH. The TyG index is a useful indicator for evaluating IR, but it is not very clear whether there is an association between the TyG index and peak GH. Therefore, this study is designed to investigate whether the TyG index was independently related to peak GH in short stature.

## Results

### Baseline characteristics of selected participants

We provide the baseline characteristics of these selected participants in the GHD and non-GHD groups in Table 1. In general, the average age of the 1095 selected participants was 10.6 ± 3.3 years old, and approximately 65.75% of them were male. No statistically significant differences were detected in height SDS, weight, systolic blood pressure (SBP), diastolic blood pressure (DBP), IGF-1 SDS, IGFBP-3, TC, HDL-C or LDL-C among the different GH groups (all *p* values > 0.05). Participants in the GHD group had higher BMI, BMI SDS, TG and TyG values (all *p* values < 0.05).

**Table1:** Clinical and biochemical characteristics.

	All	GHD	Non-GHD	*P*
Number	1095	733	362	–
Sex (male %)	720 (65.75%)	484 (66.03%)	236 (65.19%)	0.784
Age (years)	10.57 ± 3.30	10.41 ± 3.19	10.88 ± 3.49	0.027
Height (cm)	131.44 ± 17.89	130.34 ± 17.12	133.67 ± 19.18	0.004
Height SDS	− 2.85 ± 0.84	− 2.85 ± 0.85	− 2.86 ± 0.82	0.971
Body weight (kg)	31.80 ± 12.99	31.75 ± 13.13	31.91 ± 12.72	0.851
BMI (kg/m^2^)	17.57 ± 3.44	17.84 ± 3.62	17.01 ± 2.97	< 0.001
BMI SDS	− 0.05 ± 1.26	0.01 ± 1.28	− 0.16 ± 1.22	0.032
SBP (mmHg)	107.04 ± 12.30	106.57 ± 12.31	107.99 ± 12.23	0.072
DBP (mmHg)	62.67 ± 8.35	62.37 ± 8.30	63.28 ± 8.45	0.091
IGF-1 (ng/ml)	198.00 (114.75–321.00)	190.00 (120.00–283.00)	225.50(109.00–406.00)	< 0.001
IGF-1 SDS	− 1.02 (− 1.84–0.18)	− 1.02 (− 1.81–0.17)	− 1.02 (− 1.90–0.24)	0.803
IGFBP-3 (µg/ml)	4.87 ± 1.70	4.84 ± 1.82	4.93 ± 1.40	0.451
Peak GH (ng/ml)	8.92 ± 6.07	5.61 ± 2.41	15.63 ± 5.69	< 0.001
FPG (mg/dl)	85.48 ± 10.71	84.97 ± 10.81	86.54 ± 10.44	0.026
TG (mg/dl)	68.73 ± 33.80	71.89 ± 37.13	62.21 ± 24.39	< 0.001
TC (mg/dl)	147.35 ± 26.76	148.41 ± 26.70	145.15 ± 26.77	0.066
HDL (mg/dl)	52.77 ± 11.91	52.83 ± 12.39	52.66 ± 10.87	0.832
LDL (mg/dl)	264.03 ± 14.96	353.95 ± 11.14	77.55 ± 21.13	0.328
TyG index	7.89 ± 0.43	7.92 ± 0.45	7.82 ± 0.39	< 0.001
Pubertal stage				< 0.001
In prepuberty (%)	624 (56.99%)	454 (61.94%)	170 (46.96%)	
In puberty (%)	471 (43.01%)	279 (38.06%)	192 (53.04%)	

Abbreviations: Height SDS: height standard deviation scores; BMI SDS: body mass index standard deviation scores; IGF-1 SDS: insulin like growth factor-1 standard deviation scores; IGFBP-3: insulin-like growth factor-binding protein-3; Peak GH: peak growth hormone; FPG: fasting plasma glucose; TG: triglyceride; TC: total cholesterol. HDL-C: high density lipoprotein-cholesterol; LDL-C: low density lipoprotein cholesterol; TyG index: triglyceride glucose index. Normal distribution of data was presented as mean ± standard deviation; nonnormal distribution of data was presented as median (interquartile range) and categorical data using number (percentage). *P* < 0.05 is considered to be statistically significant.

### Factors associated with peak GH in the subjects

We list the results of univariate analyses in Table 2. By univariate linear regression, we found that sex, height SDS, weight, SBP, DBP, IGF-1 SDS, IGFBP-3, HDL-C and LDL-C were not associated with GH peak (all *p* values > 0.05). We also found that BMI SDS, TG, TC, and TyG were negatively associated with peak GH (all *p* values < 0.05). In contrast, univariate analysis showed that age, FPG and pubertal stage were positively correlated with peak GH (all *p* values < 0.05).

**Table 2:** Association between GH peak and different variables.

Variables	β	(95% CI)	*P* value
Age (years)	0.17	(0.06, 0.28)	0.002
Height SDS	0.03	(− 0.40, 0.46)	0.225
Body weight (kg)	− 0.01	(− 0.03, 0.02)	0.680
BMI SDS	− 0.34	(− 0.63, − 0.05)	0.020
SBP (mmHg)	0.02	(− 0.01, 0.05)	0.119
DBP (mmHg)	0.02	(− 0.02, 0.06)	0.326
IGF-1 SDS	0.16	(− 0.12, 0.45)	0.261
IGFBP-3(µg/ml)	0.13	(− 0.11, 0.36)	0.290
FPG (mg/dl)	0.05	(0.02, 0.09)	0.002
TG (mg/dl)	− 0.03	(− 0.04, − 0.02)	< 0.001
TC (mg/dl)	− 0.02	(− 0.04, − 0.01)	< 0.001
HDL (mg/dl)	− 0.01	(− 0.04, 0.02)	0.395
LDL (mg/dl)	− 0.01	(− 0.01, 0.01)	0.459
TyG index	− 2.02	(− 2.86, − 1.18)	< 0.001
**Sex**			
Male	reference		
Female	0.47	(− 0.29, 1.23)	0.226
Pubertal stage			
In prepuberty (%)	reference		
In puberty (%)	2.26	(1.55, 2.98)	< 0.001

Abbreviations: Height SDS: height standard deviation scores; BMI SDS: body mass index standard deviation scores; IGF-1 SDS: insulin like growth factor-1 standard deviation scores; IGFBP-3: insulin-like growth factor-binding protein-3; FPG: fasting plasma glucose; TG: triglyceride; TC: total cholesterol. HDL-C: high density lipoprotein-cholesterol; LDL-C: low density lipoprotein cholesterol; TyG index: triglyceride glucose index. *P* < 0.05 is considered to be statistically significant.

### The results of nonlinearity of the TyG index and peak GH

**Figure 1:**
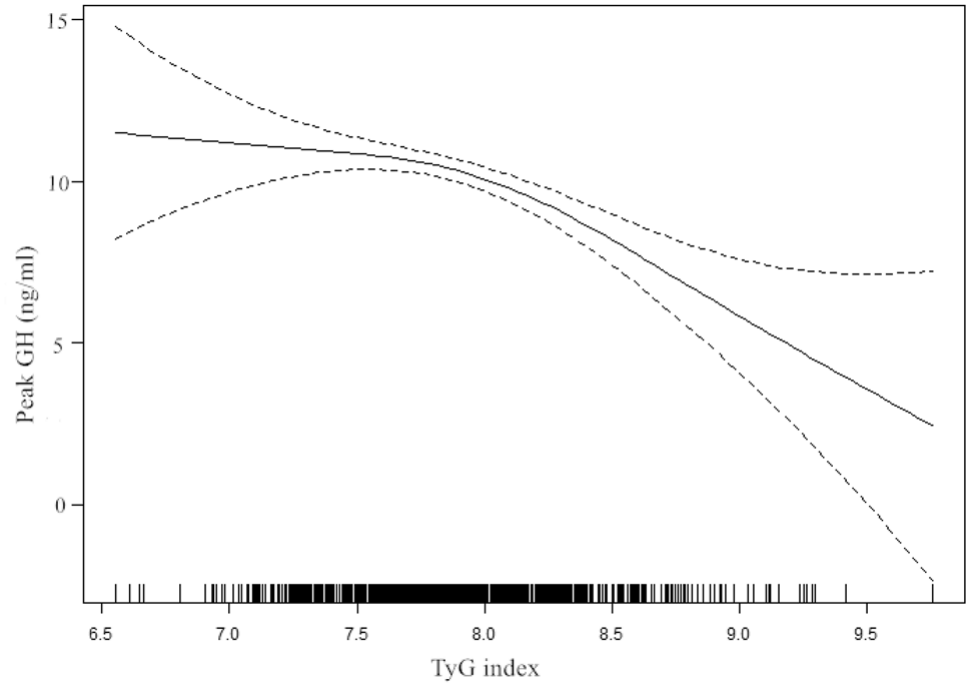
The relationship between TyG index and peak GH by smooth curve fitting. Adjustment variables: age, sex, BMI, TC, pubertal stage. BMI: body mass index, TC: total cholesterol.

In the present study, we analysed the nonlinear relationship between the TyG index and peak GH (Fig. 1). Covariate screening was done to screen for possible confounders. The screening criteria included effect factors producing a > 10% change when introducing covariates into the basic model or eliminating covariates from the regression model. The results revealed that age, sex, BMI, TC and pubertal stage met the filter criteria. The results of the smooth curve showed that the relationship between the TyG index and GH peak was nonlinear after adjusting for potential confounding factors. As shown in Table 3, we used both linear regression and two-piecewise linear regression to fit the association and select the best fit model based on P for the log likelihood ratio test.

**Table 3:** The independent association between the TyG index and peak GH.

Models	Peak GH	
	Adjusted β (95%CI)	*P* value
**Model I**		
One line slope	− 1.52 (− 2.37, − 0.66) 0.0005	< 0.001
**Model II**		
Turning point	7.8	
< 7.8 slope 1	0.25 (− 1.68, 2.17)	0.799
> 7.8 slope 2	− 2.61 (− 3.98, − 1.24)	< 0.001
LRT test	0.044	

Model I, linear analysis; Model II, non-linear analysis. LRT test, Logarithmic likelihood ratio test. (*p* value < 0.05 means Model II is significantly different from Model I, which indicates a non-linear relationship); Adjustment variables: age, sex, BMI, TC, pubertal stage. BMI: body mass index, TC: total cholesterol; *P* < 0.05 is considered to be statistically significant.

Because the P for the log likelihood ratio test was less than 0.05, we chose two-piecewise linear regression for fitting the association between the TyG index and peak GH because it can accurately represent the relationship. Using two-piecewise linear regression and a recursive algorithm, we calculated that the inflection point was 7.8. A multivariate piecewise linear regression model revealed a significant negative association between the TyG index and peak GH when the TyG index was greater than 7.8 (β − 2.61, 95% CI − 3.98, − 1.24; *P* < 0.001). However, we did not observe a significant relationship between the TyG index and GH peak when the TyG index was lower than 7.8 (β 0.25, 95% CI − 1.68, 2.17; *P* = 0.799).

## Discussion

Our findings indicate that the TyG index is negatively associated with peak GH after adjusting for other covariates. Interestingly, we found a nonlinear relationship between the TyG index and peak GH in children and adolescents with short stature. The results of this study show that a stronger association was detected when the TyG index was greater than 7.8. In contrast, we did not observe an association between the TyG index and peak GH when the TyG index was less than 7.8.

We observed that the TyG index is negatively associated with peak GH. Previous studies that directly explored the TyG index and peak GH are limited, but the TyG index is an effective indicator for evaluating IR, and the TyG index has high sensitivity for recognizing IR compared with the homeostasis model assessment of insulin resistance (HOMA-IR) index^23^. Regarding the association between IR and GH, Husbands S et al*.* reported that there is increased insulin sensitivity in young GH-deficient children^19^, but Johansson, J. O et al*.* observed that there is decreased insulin sensitivity in adults with GHD^20^. Bredella, M. A et al*.* found that peak GH was inversely associated with HOMA-IR in premenopausal women with obesity^24^*.* Our results found a negative correlation between peak GH and the TyG index, which was used to assess IR in children and adolescents with short stature. In addition to its role in promoting growth, peak GH is also an anabolic hormone that affects the metabolism of lipids and glucose, which may explain the IR with GHD^25^.

It is interesting that we revealed a nonlinear relationship between the TyG index and peak GH, suggesting that certain TyG index levels may affect the peak GH. It is well known that GHD in childhood or adulthood is associated with abnormal glucose and lipid metabolism and an increased risk of adverse cardiovascular events^17,26,27^, which indicates that the TyG index may be associated with peak GH. More importantly, the TyG index has been suggested as a reliable surrogate marker of IR, which is a substantial risk factor for adverse cardiovascular events^28–30^. The mechanisms responsible for IR in GHD are not known, but a key contributor to the markedly reduced insulin-stimulated glycogen synthase activities in GHD could be an increased availability of free fatty acids (FFAs)^31,32^. In addition, previous studies have shown that GH therapy can improve IR in GHD^33,34^.

This study demonstrates that there is an association between the TyG index and peak GH, but no causal relationship can be concluded. Our results showed that when the TyG index was greater than 7.8, a stronger association between the TyG index and peak GH was detected. This is consistent with the level of the TyG index reported in a previous study to assess the risk of IR^35^. Although the relationship between GH and IR has been well established, GH can also affect glycolipid metabolism^36^. In adipose tissue, GH facilitates lipolysis, stimulates glycerol release^37^, and inhibits lipoprotein lipase activity^38^. Increased release of FFAs from excess visceral fat may link GHD to increased glucose^36^. Furthermore, previous studies have shown that GH therapy can improve the blood lipid profile in GHD^39^.

It is well known that the secretion of GH is significantly reduced in obese individuals compared to age-matched controls^40^. Given the clear relationship between BMI and peak stimulated GH demonstrated in short stature, our findings are consistent with previous studies^6,7^. We found a negative association between BMI and peak GH in children with short stature. Our data demonstrated that the GH peak response to stimulation testing decreased with increasing BMI SDS in a large cohort of normal weight children with a range of BMIs that approximated a normal distribution (mean BMI SDS of − 0.05 ± 1.26). In addition, peak GH likely varies according to pubertal stage^41^. Our study showed a positive association between puberty and GH peak. Spontaneous and stimulated peak GH levels are higher in pubertal children than in prepubertal children. As puberty progresses, the peak GH increases, which may be related to changes in sex hormones in the body^41,42^.

However, this study has some limitations. First, because of the cross-sectional analysis, no definitive causative relationship can be inferred in the study. Second, the present findings are only based on children with short stature, and different results might be observed in other groups, such as children with obesity. Third, there may have been interobserver variability in assessing anthropometric measurements, but the anthropometric measurements were performed by one of two trained professionals. Finally, further investigation is necessary to follow up on blood lipid and glucose changes to determine whether these changes improve after GH treatment.

In conclusion, this study demonstrated that children with GHD presented an elevated TyG index compared with non-GHD children. A nonlinear relationship between the TyG index and peak GH was observed, and an increase in the TyG index was associated with a decrease in the peak GH peak in children with short stature.

## Methods

### Study population

**Figure 2:**
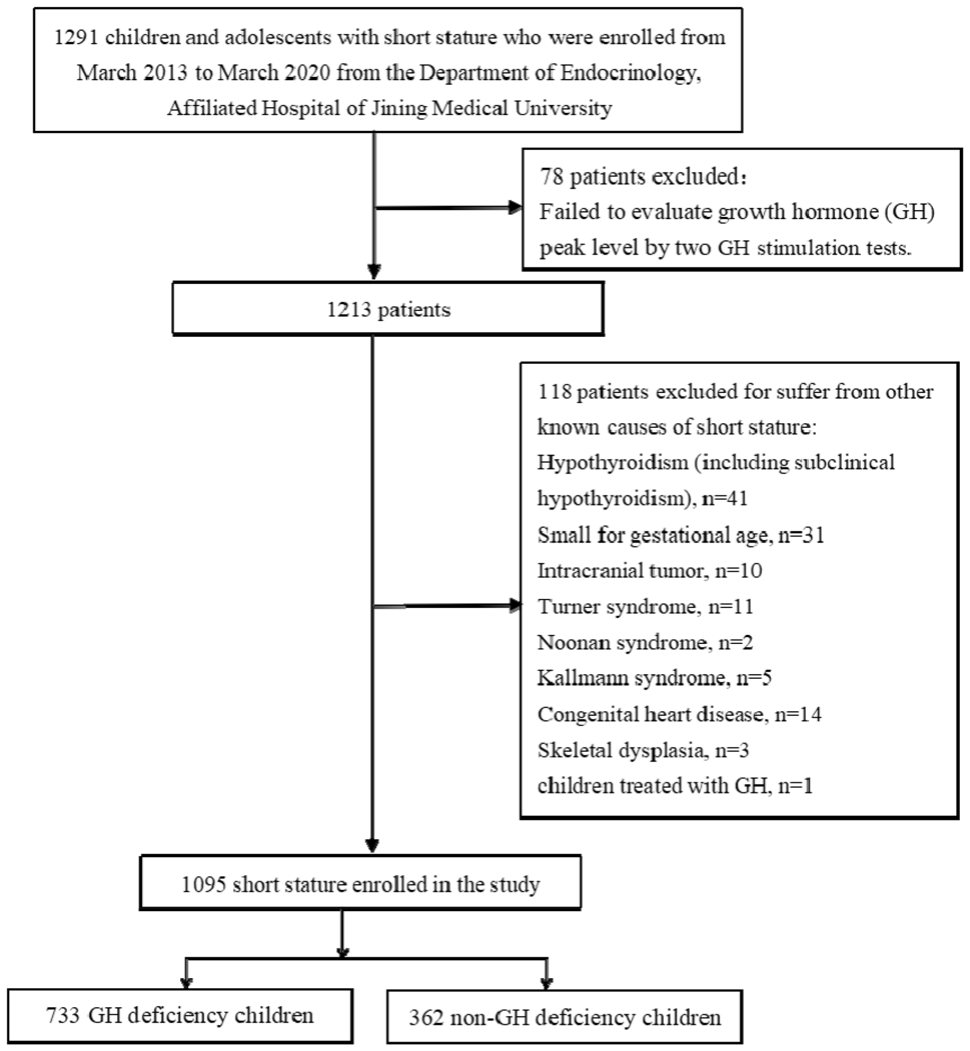
Flow chart of the study population.

The subjects were enrolled from March 2013 to March 2020 at the Department of Endocrinology, Affiliated Hospital of Jining Medical University. They are part of the GDDSD study (Growth and Development Diseases in Shandong Province: a cohort follow-up study, http://www.chictr.org.cn, ChiCTR1900026510). A total of 1095 children and adolescents with short stature (720 males and 375 females) with an average age of 10.6 ± 3.3 years were enrolled. All enrolled children were assessed for GHD and non-GHD based on the GH peak level in provocation tests. Children with GH peaks below 10 ng/mL in two different provocation tests were classified as GHD^43^. The subjects with a height SDS lower than or equal to − 2 SD after adjusting for age and sex, an appropriate birth weight for gestational age, and who completed two GH stimulation tests were included in the study. The exclusion criteria included children with skeletal dysplasia, thyroid dysfunction or other known causes of short stature, including Noonan syndrome, Turner syndrome, or having been small for gestational age. In addition, children treated with medication interfering with GH secretion or its action were also excluded. The flow chart of the study selection process is shown in Fig. 2.

### Ethics

This proposal was reviewed and approved by the Human Ethics Committee of the Affiliated Hospital of Jining Medical University (JYFY-2015-019) and all methods were performed in accordance with the guidelines of the Declaration of Helsinki. Informed consent was obtained from all participants or their parents.

### Data collection

All of the data were collected by reviewing the medical records of the medical centre. The height was measured to the nearest 0.1 cm without shoes using a stadiometer (Nantong Best Industrial Co, Ltd., Jiangsu; China). The weight was measured by an electronic scale to the nearest 0.1 kg (Wuxi Weigher Factory Co, Ltd., Jiangsu; China) while the individual was wearing light clothes without shoes. In addition, height and weight were measured by a designated individual using the same measuring instrument in the morning. Height SDS was expressed using the growth curve of Chinese children as a reference^44^. The body mass index (BMI) was calculated as the ratio of the weight divided by the square of the height in metres, and the BMI SDS was calculated according to the normal children reference^45^. Puberty stage was evaluated by one of two pretrained physicians during a physical examination, based on the Tanner stages^46^. The following criteria can be considered prepubescent: boys with no pubic hair and testicular volume less than 4 mL and girls with no pubic hair and no breast development.

Fasting blood samples were obtained from all subjects for measuring laboratory parameters. Two stimulating tests were performed for GH (500 mg of levodopa for those weighing more than 30 kg; 250 mg of levodopa for those weighing less than 30 kg, orally and 0.1 U/kg insulin, subcutaneously). Blood samples were collected at 0, 30, 60, 90, and 120 min after administration to obtain serum GH concentrations at each time point. A chemiluminescence method was used to assess GH concentration (ACCESS2, Beckman Coulter; USA). It had intra- and interassay CVs of 3.5% and 5.8%, respectively. Insulin-like growth factor-1 (IGF-1) and IGF binding protein-3 (IGFBP-3) were measured by a chemiluminescence assay (DPC IMMULITE 1000 analyser, SIEMENS, Germany) that had intra- and interassay CVs of 3.0% and 6.2% for IGF-1 and 4.4% and 6.6% for IGFBP-3, respectively. Fasting plasma glucose (FPG), lipid profiles (triglycerides (TG), total cholesterol (TC), high-density lipoprotein-cholesterol (HDL-C), and low-density lipoprotein-cholesterol (LDL-C)) were determined by an autobiochemical analyser (Cobas c702, Roche; Shanghai, China). The interassay coefficient of variation of the TG assay was 8.3%, TC was 3.3%, HDL-C was 10.0%, LDL-C was 10.0% and FPG was 3.0%. The TyG index was obtained as ln [fasting TG (mg/dl) × FPG (mg/dl)/2]^23^. The IGF-1 SDS was calculated based on IGF-1 levels matched to the age and sex of healthy children and adolescents identified in Japan^47^. All methods were performed in accordance with the relevant guidelines and regulations.

### Statistical analysis

In this study, our presentation of continuous variables was based primarily on whether they were normally distributed. If it was a normal distribution, we present continuous variables as the mean ± standard, and vice versa as the median (interquartile). Categorical variables were expressed as frequencies or percentages. We used χ^2^ (categorical variables), Student’s t test (normal distribution), or the Kruskal–Wallis H test (skewed distribution) to test for differences among the GHD and non-GHD groups. Univariate analysis was used to assess whether the TyG index and other variables were associated with the GH peak. Multiple linear regression adjusted for potential confounding factors was used to further analyse the independent association between the TyG index and GH peak. A smooth curve fitting was used to explore the relationship between the TyG index and GH peak. Finally, a multivariate piecewise linear regression was further used to examine the threshold correlation of the TyG index and GH peak according to the smooth curve fit. A two-tailed *P* < 0.05 was considered statistically significant in all analyses. Statistical analysis was performed with R 3.4.3 (https://www.R-project.org) and EmpowerStats (https://www.empowerstats.com, X&Y Solutions, Inc. Boston MA).

## References

[1] Yue D, Miller MR, Clarson CL (2019). Evaluation of referrals for short stature: A retrospective chart review. Paediatr. Child Health.

[2] Wilson JR, Utz AL, Devin JK (2016). Effects of gender, body weight, and blood glucose dynamics on the growth hormone response to the glucagon stimulation test in patients with pituitary disease. Growth Horm. IGF Res..

[3] Lee J (2013). Influence of body mass index on the growth hormone response to provocative testing in short children without growth hormone deficiency. J. Korean Med Sci..

[4] Ho KY (1987). Effects of sex and age on the 24-hour profile of growth hormone secretion in man: Importance of endogenous estradiol concentrations. J. Clin. Endocrinol. Metab..

[5] Smotkin-Tangorra M, Anhalt H, Ten S (2006). Growth hormone and premature atherosclerosis in childhood obesity. J. Pediatr. Endocrinol Metab..

[6] Yang A (2019). Impact of BMI on peak growth hormone responses to provocative tests and therapeutic outcome in children with growth hormone deficiency. Sci. Rep..

[7] Stanley TL, Levitsky LL, Grinspoon SK, Misra M (2009). Effect of body mass index on peak growth hormone response to provocative testing in children with short stature. J. Pediatr. Endocrinol. Metab..

[8] Misra M (2008). Lower growth hormone and higher cortisol are associated with greater visceral adiposity, intramyocellular lipids, and insulin resistance in overweight girls. Am. J. Physiol. Endocrinol. Metab..

[9] Park G (2020). Triglyceride glucose index is a useful marker for predicting subclinical coronary artery disease in the absence of traditional risk factors. Lipids Health Dis..

[10] Xie H, Song J, Sun L, Xie X, Sun Y (2020). Independent and combined effects of triglyceride-glucose index on prehypertension risk: A cross-sectional survey in China. J. Hum. Hypertens.

[11] Won K (2020). Triglyceride glucose index is an independent predictor for the progression of coronary artery calcification in the absence of heavy coronary artery calcification at baseline. Cardiovasc. Diabetol..

[12] Hattori S (2020). Omarigliptin decreases inflammation and insulin resistance in a pleiotropic manner in patients with type 2 diabetes. Diabetol. Metab. Syndr..

[13] Zhao H, Wang G, Zhang M, Tong W, Zhang Y (2011). Prehypertension and insulin resistance among Mongolian people, inner Mongolia, China. Blood Pressure.

[14] American DA (1998). Consensus development conference on insulin resistance: 5–6 November 1997. Diabetes Care.

[15] Fernando GR (2010). The product of triglycerides and glucose, a simple measure of insulin sensitivity. Comparison with the euglycemic-hyperinsulinemic clamp. J. Clin. Endocrinol. Metab..

[16] Vijayakumar A, Yakar S, LeRoith D (2011). The intricate role of growth hormone in metabolism. Front. Endocrinol..

[17] Liang S, Xue J, Li G (2018). Effects of recombinant human growth hormone administration on cardiovascular risk factors in obese children with relative growth hormone deficiency. Lipids Health Dis..

[18] Rabinowitz D, Zierler KL (1963). A Metabolic regulating device based on the actions of human growth hormone and of insulin, singly and together, on the human forearm. Nature.

[19] Husbands S, Ong KKL, Gilbert J, Wass JAH, Dunger DB (2001). Increased insulin sensitivity in young, growth hormone deficient children. Clin. Endocrinol (Oxf)..

[20] Johansson JO, Fowelin J, Landin K, Lager I, Bengtsson BA (1995). Growth hormone-deficient adults are insulin-resistant. Metabolism..

[21] Salomon F, Cuneo R, Sonksen PH (1993). Insulin sensitivity and insulin resistance in growth-hormone-deficient adults. Horm. Res..

[22] Vogelzangs N (2020). Metabolic profiling of tissue-specific insulin resistance in human obesity: Results from the diogenes study and the maastricht study. Int. J. Obes..

[23] Simental-Mendía LE, Rodríguez-Morán M, Guerrero-Romero F (2008). The product of fasting glucose and triglycerides as surrogate for identifying insulin resistance in apparently healthy subjects. Metab. Syndr. Relat. Disord..

[24] Bredella MA (2009). Peak growth hormone-releasing hormone-arginine-stimulated growth hormone is inversely associated with intramyocellular and intrahepatic lipid content in premenopausal women with obesity. J. Clin. Endocrinol. Metab..

[25] Murray RD (2002). Low-dose GH replacement improves the adverse lipid profile associated with the adult GH deficiency syndrome. Clin Endocrinol (Oxf)..

[26] De Leonibus C (2016). Growth hormone deficiency in prepubertal children: Predictive markers of cardiovascular disease. Horm. Res. Paediat..

[27] Lanes R (2016). Cardiovascular risk in growth hormone deficiency. Endocrin. Metab. Clin..

[28] Guerrero-Romero F (2016). Fasting triglycerides and glucose index as a diagnostic test for insulin resistance in young adults. Arch. Med. Res..

[29] Vasques AC (2011). TyG index performs better than HOMA in a Brazilian population: A hyperglycemic clamp validated study. Diabetes Res. Clin. Pract..

[30] Hanley AJG (2002). Homeostasis model assessment of insulin resistance in relation to the incidence of cardiovascular disease: The San Antonio heart study. Diabetes Care.

[31] Bjorntorp P (1991). Metabolic implications of body fat distribution. Diabetes Care..

[32] Christopher M, Hew FL, Oakley M, Rantzau C, Alford F (1998). Defects of insulin action and skeletal muscle glucose metabolism in growth hormone-deficient adults persist after 24 months of recombinant human growth hormone therapy. J. Clin. Endocrinol. Metab..

[33] Maison P (2004). Impact of growth hormone (GH) treatment on cardiovascular risk factors in GH-deficient adults: A metaanalysis of blinded, randomized, placebo-controlled trials. J. Clin. Endocrinol. Metab..

[34] Svensson J, Fowelin J, Landin K, Bengtsson B, Johansson J (2002). Effects of seven years of GH-replacement therapy on insulin sensitivity in GH-deficient adults. J. Clin. Endocrinol. Metab..

[35] Vieira-Ribeiro SA (2019). The TyG index cutoff point and its association with body adiposity and lifestyle in children. J. Pediatr. (Rio J)..

[36] Møller N, Jørgensen JO (2009). Effects of growth hormone on glucose, lipid, and protein metabolism in human subjects. Endocr. Rev..

[37] Djurhuus CB (2004). Additive effects of cortisol and growth hormone on regional and systemic lipolysis in humans. Am. J. Physiol. Endocrinol. Metab..

[38] Ottosson M (1995). Growth hormone inhibits lipoprotein lipase activity in human adipose tissue. J. Clin. Endocrinol. Metab..

[39] Chen M (2018). Effect of recombinant human growth hormone therapy on blood lipid and carotid intima-media thickness in children with growth hormone deficiency. Pediatr. Res..

[40] Savastano S, Somma CD, Barrea L, Colao A (2014). The complex relationship between obesity and the somatropic axis: The long and winding road. Growth Horm. IGF Res..

[41] Marin G (1994). The effects of estrogen priming and puberty on the growth hormone response to standardized treadmill exercise and arginine-insulin in normal girls and boys. J. Clin. Endocrinol. Metab..

[42] Rose SR (1991). Spontaneous growth hormone secretion increases during puberty in normal girls and boys. J. Clin. Endocrinol. Metab..

[43] Growth Hormone Research Society (2000). Consensus guidelines for the diagnosis and treatment of growth hormone (GH) deficiency in childhood and adolescence: Summary statement of the GH research society. GH research society. J. Clin. sEndocrinol. Metab..

[44] Li H, Ji CY, Zong XN, Zhang YQ (2009). Height and weight standardized growth charts for Chinese children and adolescents aged 0 to 18 years. Chin. J. Pediatr..

[45] Li H, Ji CY, Zong XN, Zhang YQ (2009). Height and weight standardized growth charts for Chinese children and adolescents aged 0 to 18 years. Chin. J. Pediatr..

[46] Wright CM (2012). Can we characterise growth in puberty more accurately? Validation of a new puberty phase specific (PPS) growth chart. Arch. Dis. Child..

[47] Isojima T (2012). Standardized centile curves and reference intervals of serum insulin-like growth factor-I (IGF-I) levels in a normal Japanese population using the LMS method. Endocr. J..

